# Cases from Practice

**Published:** 1873-11

**Authors:** J. W. Brooks

**Affiliations:** Chicago


Article III.—*Cases from Practice.* By J. W. Brooks, M.D., Chicago.

Case 1. Sept. 1st, 1873, was invited at 1:30 o'clock P. M., to visit Mrs. L., a multipara, 24 years of age, under the care of a midwife, at No. —, —— Avenue. Said to have been in labor from twenty to twenty-four hours. On reaching the bedside found a peculiar spasmodic condition of the uterus; pulse at the wrist almost imperceptible; lips blue; incessant jactitation; hurried, grunting respiration; cool sweat over the body; great anxiety.

Administered McMunn's elixir of opium, and sent for professional assistance, to which Dr. McWilliams responded. The system in no way responded to the opiate. The child was forthwith removed with the forceps, and a free flow of uncoagulated blood followed, which did not coagulate afterward. The rupture appeared to be at the lower part of the uterus, near to the vagina. (Could not obtain an autopsy.) The hand passed readily into the abdominal cavity. The uterus was found well contracted below and to the left side. There was no further external hemorrhage. Stimuli were administered without result, the patient continued to sink, and died at 4:10 P. M. the same day. I learned from the attendants that the membranes gave way at 8 A. M. with a snap and a gush of waters, and that nothing flowed afterward till the child was removed, and that it was at 8 A. M. that the restlessness and painful breathing commenced. I think that the rupture commenced at that hour, and that the blood that passed off was the accumulation of several hours. *Quære:* Is it usual for the blood under like circumstances not to form a coagulum?

I omitted to mention that there was a remarkably distinct varicosity of the veins of the vulva, extending some distance up the vagina.

CASE 2. Sept. 1st, 1873, at 8 A. M., was requested to visit Mr. N., a resident of Forty-ninth street, in haste. On arrival, found an atlo-axoid luxation incomplete, and a partial bilateral dislocation of the fifth cervical vertebra. He is a well developed, muscular man, of about 28 years. His sufferings were intense—perhaps we may say agonizing. He was numb from the axis down; had been very uneasy from the time of the injury, at 6 P. M. the preceding evening, to the then present time, and unable to move. It should have been stated that he was picked up unconscious, and returned only gradually to consciousness. The injuries had occurred on this wise: He was riding with his lady in a buggy, and when about to turn a street corner, the horse moving rapidly, he turned to look behind him, and in the act was tossed from his buggy, alighting on the top of his head apparently, and making a complete somersault. The chin was raised a little, turned to the left, and immovably fixed; the face cool, pale and bluish. He being half recumbent, the writer stood directly behind him, while an assistant placed a hand on each shoulder and there held firmly downwards. I then grasped the base of the skull on either side with a hand and made a counter-extension, and a gentle and forward rotary motion, and the atlo-axoid articulation was restored with a snap, attended with great relief. Still retaining my position, I placed one thumb on the spinous process of the fifth cervical vertebra and the fore and middle finger on the projecting portion of the side of the vertebra; pressing firmly with these, and with the other hand carefully rotating, slightly, and making counter-extension to the best of my ability, while my assistant pressed very firmly down on the shoulders, the vertebra was thus returned to its normal position with an audible click. Sensation and motion were speedily restored. A napkin wrung from warm water was applied from the head to the first dorsal vertebra for forty-eight hours. September 3d, the patient was convalescing rapidly.

CASE 3. Sept. 26th, 1872, was requested hurriedly to visit F. B., a delicate, pale boy of 8 or 9 years. On arriving, learned that on the previous evening, while playing at somersault in his father's parlor, he had injured his neck. Examination revealed a sub-atlo-axoid luxation. The chin was directed a little upward and to the right; the head was immobile; there was some numbness below the seat of injury, in all of the members of the body; he was exceedingly nervous; there was no intense pain, but, as he expressed it, "it hurt all the time;" with a pale, damp, cool skin. With an assistant to steady and hold down the shoulders, and standing directly behind him (he being half recumbent), with a hand on either side of the base of the cranium, and the forefinger separated from the middle finger so that one forefinger of each hand would come before the articulation, and the middle finger of each hand behind, I proceeded to rotate carefully, and at the same time to make counter-extension; presently an audible click announced the return of the parts to their normal position. Convalescence was soon established.

